# Evaluation of miRNA Profile and Its Relationship with Metabolic Disorders in Obese and Pre-Obese Patients

**DOI:** 10.3390/cimb47040280

**Published:** 2025-04-17

**Authors:** Kürşat Kargün, Erhan Aygen, Mehmet Fatih Ebiloğlu, Naci Ömer Alayunt, Lütfiye Kadıoğlu Dalkılıç

**Affiliations:** 1 Department of Nursing, Faculty of Health Sciences, Fırat University, Elazig 23119, Turkey; 2 Department of General Surgery, Faculty of Medicine, Firat University, Elazig 23119, Turkey; eaygen@firat.edu.tr; 3 General Surgery Department, Fethi Sekin City Hospital, Elazığ 23280, Turkey; mehmetfatihebiloglu@gmail.com; 4 Medical Biochemistry Department, Faculty of Medicine, Siirt University, Siirt 56100, Turkey; naci.alayunt@siirt.edu.tr; 5 Molecular Biology and Genetics Program, Department of Biology, Faculty of Science, Firat University, Elazig 23119, Turkey; lkdalkilic@firat.edu.tr

**Keywords:** fludeim, miRNA, pre-obese, obese

## Abstract

Obesity is a growing global public health concern, with its prevalence rapidly increasing in Turkey, leading to severe consequences. Genetic factors, particularly mutations in structural genes and microRNAs (miRNAs) involved in gene expression regulation have been widely investigated in obesity research. This study aimed to explore the role of obesity-associated miRNAs and their potential interaction with vascular response alterations. A total of 60 obese and pre-obese patients and 26 age- and sex-matched healthy controls from the General Surgery Department of Fırat University Medicine School were included. The expression levels of 93 miRNAs were analyzed in 86 samples using the Fluidigm Biomark RT-PCR system, with 5S RNA as the housekeeping gene. Significant differences were observed in weight, BMI, cholesterol, triglyceride levels, and lymphocyte counts between the groups (*p* < 0.0001). Several miRNAs, including hsa-miR-148a-3p, hsa-miR-503-3p, hsa-miR-34a-5p, and hsa-miR-199a-3p, were significantly downregulated in obese patients. Additionally, gender-specific differences in miRNA expression were identified. These findings indicate that miRNAs play a crucial role in obesity pathophysiology and could serve as potential biomarkers and therapeutic targets for obesity treatment.

## 1. Introduction

Obesity and weight gain are defined as excessive fat accumulation leading to health problems [1]. Adipocytes are the main component of adipose tissue, playing a critical role in the regulation of predisposition to obesity [2]. According to data from the World Health Organization (WHO), the incidence of obesity has increased since 1975, and it is estimated that one in five people will be obese by 2025 [1]. Obesity is a major risk factor for chronic diseases such as diabetes, cancer, and cardiovascular diseases, and it is considered a concerning public health issue due to its increasing prevalence [1].

Obesity results from an imbalance between energy intake and expenditure and has a complex etiology where metabolic, genetic, hormonal, environmental, and psychological factors are involved [3]. The decrease in basal metabolic rate and lack of physical activity when aging may lead to obesity by further increasing energy imbalance. It has been reported that obesity rates are higher in women, particularly in older age groups, compared to men [3,4]. It has been suggested that the prevalence of obesity is almost two-fold higher among women than men in Turkey [5,6]. The sedentary lifestyle has become increasingly common, and it has facilitated access to foods with high fat and sugar content with industrialization and urbanization. Increased fast-food consumption and irregular eating habits are among the key factors triggering obesity [7,8,9].

Genetic studies have shown that genetic factors are involved in appetite, fat distribution, energy metabolism, and adipocyte differentiation [10]. Familial predisposition is the most relevant one among genetic components of obesity [11]. In recent years, the effects of miRNAs on obesity pathogenesis have drawn attention due to their roles in the post-transcriptional regulation of gene expression [12,13]. miRNAs are non-coding RNA molecules 18–22 nucleotides in length, which are involved in many biological and pathological processes by regulating gene expression negatively [14,15]. Mature miRNAs inhibit protein translation or degrade mRNA by binding to target mRNAs [14,15,16]. It has been shown that the levels of miRNAs expressed in adipocytes are changed depending on obesity development [17]. For instance, it has been reported that miR-122, miR-143, and miR-27a are associated with obesity, playing significant roles in lipid metabolism and adipocyte differentiation [18,19,20,21,22,23,24].

The interaction of miRNAs with genetic mutations and environmental factors suggests that they function as fine-tuning regulators in obesity pathogenesis [25,26]. There are also several studies suggesting that obesity is associated with miRNAs, emphasizing the role of miRNAs in disease pathogenesis, progression, and/or inhibition [27]. In addition, there are more than 2600 miRNAs identified in humans; it has been suggested that the dysregulated expression of an individual miRNA or entire miRNA network may contribute to disease pathogenesis and/or progression, indicating the complexity of gene regulation processes [28]. There are several studies that confirm the association between miRNAs (miR-122, miR-143, miR-27a, etc.) and obesity [29,30,31]. In some studies, it has been shown that miRNAs may be up-regulated or downregulated in association with obesity [2,32].

Given their ability to regulate gene expression, it has been reported that miRNAs play roles in a range of pathophysiological processes, including hypertension, obesity, and atherosclerosis [33,34,35]. miRNAs control cellular processes and regulate developmental, metabolic, cellular, and immune system functions by suppressing target gene expression. The relationship between obesity and miRNAs has been intensively studied in recent years, and it is thought that miRNAs may play a key role in elucidating the molecular mechanisms underlying obesity [36,37]. The cellular processes regulated by miRNAs include inflammation, adipose tissue homeostasis, insulin resistance, and cell proliferation. Furthermore, miRNAs have a key role in the development of metabolic diseases associated with obesity. For example, there is a growing number of studies on the effects of miRNAs such as hsa-miR-34a, hsa-miR-148a, and hsa-miR-503 on obesity-related genetic and metabolic processes [38,39]. However, it is unclear how miRNAs interact with the pathophysiology of obesity and which miRNAs are specifically involved.

In the literature, there are several studies suggesting a relationship between obesity and genetic factors [11]. In this study, we evaluated the effects of miRNAs on the development of obesity and pre-obesity. It also aimed to assess the expression profiles of miRNAs in obese and pre-obese patients; improve understanding of the genetic and physiological effects of miRNAs; and investigate their potential as obesity biomarkers. miRNAs are considered important regulators of many metabolic activities, but their role in obesity-related tissue and organ damage is not fully understood; thus, the determination of miRNA levels may provide significant insights into the prevention, diagnosis, prognosis, and identification of drug target molecules for the disease. This may provide a significant contribution to the development of effective methods that can be used to prevent obesity via early intervention and mortality in clinical conditions associated with obesity.

## 2. Materials and Methods

### 2.1. Study Sample

The study included 60 patients diagnosed with obesity (*n* = 30) and pre-obesity (*n* = 30) by the General Surgery Department of Fırat University, Medicine School, and 26 age- and sex-matched healthy individuals without a history of hypertension, chronic disorders, acute infection, or medication as controls. The inclusion criteria for the diagnosis of obesity and pre-obesity were based on the body mass index (BMI) classification system. According to the World Health Organization (WHO) guidelines, individuals with a BMI of 25–29.9 kg/m^2^ were classified as pre-obese, while those with a BMI of 30 kg/m^2^ or higher were classified as obese. In the patient and control groups, personal family histories were obtained from all subjects. Demographic characteristics, including age, gender, and body mass index, were recorded for all subjects. Blood samples from patient and control groups were collected into EDTA tubes for genetic testing. These samples were treated with the RNA stabilization reagent and stored at −20 °C in a freezer. Then, after total RNA isolation, nucleic acid was isolated using the Zymo Total RNA Isolation Kit. Subsequently, total RNA was converted into cDNA and stored at −20 °C. Samples were kept at −20 °C until miRNA profiling. miRNA profiles were determined using a Fluidigm Biomark RT-PCR instrument. Threshold cycle (Ct) values were obtained for each sample, and each miRNA was analyzed by the relative quantification statistical analysis method; 5S RNA was preferred as the housekeeping gene in the analysis for normalization. Raw data are provided as Supplementary Materials File S1. Additional blood samples (5 cc) were collected in biochemistry tubes and centrifuged. The sera were stored at −20 °C until lipid profile analysis (total cholesterol, HDL, LDL, and triglycerides). A complete blood count (CBC), including hemoglobin, neutrophils, lymphocytes, and platelets, was also performed.

### 2.2. Statistical Methods

Statistical analysis was performed using SPSS^®^ for Windows, v. 15.0. The results are presented as the mean ± standard deviation. Analyses were performed on the Qiagen Data Analysis Center website (http://www.qiagen.com/us/shop/genes-and-pathways/data-analysis-center-overview-page/, accessed on 23 October 2024). RT-PCR results (Ct values) were calculated using the 2^−ΔΔCt^ method. One-way analysis of variance (ANOVA) was used to evaluate the data. Tukey’s post hoc test was employed for pairwise group comparisons, and the Benjamini and Hochberg methods were utilized to control False Discovery Rates (FDRs) in multiple comparisons. A *p*-value < 0.05 was considered statistically significant.

## 3. Results

### 3.1. Demographic and Clinical Data

Significant differences were observed between the obese, pre-obese, and control groups regarding weight (*p* < 0.0001) and BMI (*p* < 0.0001). In addition, as seen in Table 1, significant differences were observed between the groups in terms of cholesterol, triglyceride, and lymphocyte levels.

### 3.2. miRNA Expression Profiles

As shown in Table 2, Table 3, Table 4, Table 5, Table 6 and Table 7, the expression levels of miRNAs such as hsa-miR-148a-3p, hsa-miR-503-3p, and hsa-miR-34a-5p were decreased in obese and pre-obese patients. There was an increase in the hsa-miR-519b-3p expression (Table 6 notably shows the negative regulation of hsa-miR-148a-3p expression (*p* < 0.000365)), which suggests that it may play an inhibitory role in the pathophysiology of obesity.

These findings indicate that the state of obesity is associated with significant differences at both clinical and genetic levels. In particular, it was found that miRNA expression profiles varied according to the state of obesity and gender differences. These results support the role of miRNAs in the molecular mechanisms of obesity, indicating their potential use as biomarkers for obesity.

## 4. Discussion

In this study, we evaluated the effects of obesity and pre-obesity on clinical and biochemical characteristics and miRNA expression profiles. Our findings indicate that obesity does not only lead to physical and biochemical changes but also to differences at the molecular level.

### 4.1. Clinical and Biochemical Assessments

The significantly higher weight and body mass index (BMI) values in the obese group compared to the control group support the physiological effects of obesity. The increase in total cholesterol and triglyceride levels demonstrates that obesity is directly associated with metabolic risk factors [40]. Our findings are in agreement with previous studies that investigated the relationship between obesity and risk for cardiovascular diseases. The increased lymphocyte count is associated with the recognition of obesity as a low-grade inflammatory state. In the literature, it has been reported that inflammatory responses are enhanced in obese individuals [41]. This study supports this finding.

### 4.2. miRNA Expression Analyses

In this study, significant changes were observed in the expression of certain miRNAs in obese and pre-obese individuals. Particularly, the decreased expression of hsa-miR-148a-3p and hsa-miR-503-3p in obese individuals compared to the control group indicates their potential roles in the molecular mechanisms of obesity. It has been shown in the literature that hsa-miR-148a-3p is associated with genes regulating adipose tissue metabolism and insulin signaling pathways [42]. The decreased expression of hsa-miR-124-3p in pre-obese individuals emphasizes the possible role of this miRNA in the transition to obesity. The decreased expression of hsa-miR-488-3p and hsa-miR-384 in obese males is in agreement with studies examining the effects of gender differences on obesity at the molecular level [43]. The finding of decreased hsa-miR-503-3p expression in obese females indicates that hsa-miR-503-3p plays a significant role in adipocyte differentiation and energy metabolism. These findings indicate that gender-related differences influence obesity-related miRNA expression profiles.

Statistical analysis between obese individuals (both obese and pre-obese) and the control group showed a significant decrease in hsa-miR-148a-3p expression. This miRNA plays a fundamental role in regulating lipid metabolism, inflammation, and glucose homeostasis. A reduction in hsa-miR-148a-3p expression is known to increase cholesterol synthesis and lipid accumulation [44]. The decreased levels of this miRNA in obese individuals may reflect the metabolic disorders caused by obesity. Additionally, the expression of hsa-miR-503-3p was reduced in obese patients. This miRNA is involved in cell cycle regulation and angiogenesis, with decreased levels potentially impairing adipose tissue vascularization and energy metabolism [45]. The elevated triglyceride levels observed in this study support this. These findings suggest the involvement of hsa-miR-148a-3p and hsa-miR-503-3p in obesity pathophysiology, specifically affecting lipid metabolism and vascular function. These findings suggest that hsa-miR-148a-3p and hsa-miR-503-3p play important roles in the pathophysiology of obesity and that these miRNAs can be used as biomarkers. Overall, changes in the expression of these miRNAs can serve as molecular markers for obesity and related metabolic disorders, with future research critical in evaluating them as therapeutic targets.

In the comparison between obese and control groups, a decrease in the expression of hsa-miR-34a-5p was observed, which plays a significant role in oxidative stress and cellular aging. This reduction may indicate increased cellular stress responses and mitochondrial dysfunction in obesity. The literature suggests that decreased hsa-miR-34a-5p expression can impair adipocyte differentiation and insulin sensitivity [46]. Zheng et al. (2022) reported similar downregulation in the context of liver damage [47]. In a preliminary study on asthmatic patients, Mirra et al. found significantly lower expression levels of hsa-miR-34a-5p, hsa-miR-181a-5p, and hsa-miR-146a-5p in both obese and healthy individuals compared to asthmatic patients, suggesting that these miRNAs play a specific role in regulating lung inflammatory responses [48]. Furthermore, the reduced expression of hsa-miR-199a-3p indicates disturbances in energy metabolism and inflammation regulation. The up-regulation of hsa-miR-199a-3p has been linked to obesity in children [49]. Reduced levels of hsa-miR-148a-3p were also noted in this study, reflecting impairments in lipid homeostasis. This miRNA regulates cholesterol synthesis and adipose tissue inflammation [50]. Similarly, hsa-miR-34a-3p plays a role in oxidative stress and inflammatory responses, with its reduction contributing to inflammation and energy imbalance in obesity [51]. The decreased expression of hsa-miR-143-3p, which regulates adipocyte differentiation, may suggest impaired adipose tissue functionality [52]. These findings support previous studies by Jordan et al. (2011) and Goedeke et al. (2014), indicating that alterations in miRNA profiles in obesity are associated with metabolic and inflammatory disorders [44,51]. Specifically, reductions in hsa-miR-34a-5p, hsa-miR-143-3p, and hsa-miR-148a-3p could contribute to obesity’s primary mechanisms, such as adipocyte dysfunction and energy imbalance. The changes in miRNA expression levels offer potential biomarkers for diagnosing and treating obesity.

The decreased expression of hsa-miR-124-3p in the analysis between the groups in the present study emphasizes the role of this miRNA in energy metabolism and inflammatory processes. In the literature, the decreased expression of hsa-miR-124-3p indicates insulin resistance and increased metabolic stress [53]. The decreased expression of this miRNA in pre-obese individuals may be an early sign of metabolic disorders. It has been reported that hsa-miR-124-3p plays a significant role in regulating inflammation, and its reduced expression contributes to adipocyte dysfunction, while other studies have suggested that the decrease in hsa-miR-124-3p expression may be associated with insulin resistance, particularly affecting insulin signaling pathways [52,54].

A comparison of miRNA profiles in obese women and men revealed a significant decrease in hsa-miR-218-1-3p expression, which is involved in energy homeostasis, inflammation, and vascular regulation. This decrease may indicate metabolic differences across genders, influenced by factors such as hormone levels and fat distribution. Studies have linked reduced hsa-miR-218-1-3p expression to vascular inflammation, metabolic syndrome, and insulin resistance [55,56]. Its lower expression in obese men suggests a higher predisposition to vascular dysfunction and metabolic disorders. Given its role in insulin signaling and glucose metabolism, hsa-miR-218-1-3p may serve as a biomarker for gender-specific metabolic differences in obesity, potentially guiding individualized treatment strategies.

The statistical analysis of obese vs. control males revealed significant miRNA expression changes. The increased levels of hsa-miR-519b-3p may contribute to impaired adipose tissue homeostasis, neuroendocrine regulation, and disrupted energy balance by affecting genes like PTEN and FOXO1 [57,58]. Decreased levels of hsa-miR-488-3p and hsa-miR-148a-3p were linked to impaired glucose metabolism, insulin sensitivity, and hepatic lipid regulation, increasing the risk of hepatic steatosis [59,60]. Reduced hsa-miR-34a-3p and hsa-miR-877-3p levels were associated with inflammatory processes and vascular dysfunction. These findings highlight the role of miRNAs in metabolic and inflammatory pathways in obese males. Gender-specific miRNA expression analysis may offer insights into obesity-related complications and support personalized treatment strategies [61].

The statistical analysis of obese vs. control females revealed that hsa-miR-503-3p plays a key role in metabolism, inflammation, and angiogenesis. Its decreased expression may contribute to adipose tissue inflammation, vascular dysfunction, and metabolic disorders. This alteration may impair adipocyte differentiation, increase fat storage, and trigger metabolic stress [62]. hsa-miR-503-3p is linked to endothelial function and glucose metabolism, with sex-specific differences potentially influencing its regulation. Studies have associated its reduction with vascular inflammation and adipocyte dysfunction, suggesting that it could act as a biomarker for obesity-related metabolic and inflammatory processes [61,62]. Its role in cardiometabolic risk makes it a potential therapeutic target. miRNAs play a critical role in obesity by regulating the target genes involved in lipid metabolism, insulin signaling, and inflammation. This study identified significant obesity-related miRNAs and their target genes, highlighting their regulatory roles in metabolic and inflammatory processes (Table 8).

hsa-miR-148a-3p regulates energy metabolism by targeting PPARGC1A (Peroxisome proliferator-activated receptor gamma coactivator 1-alpha), FTO, and modulating adipogenesis and leptin (LEP) signaling, thus impacting energy metabolism [63]. The reduction in hsa-miR-503-3p affects vascular responses and tissue oxygenation by suppressing VEGFA (vascular endothelial growth factor A) and FGF2, contributing to metabolic dysfunctions in obese females [64]. hsa-miR-34a-5p regulates lipogenesis and adipocyte differentiation through SIRT1 (sirtuin 1) and PPARG suppression, linking it to visceral fat accumulation and insulin resistance [65]. The altered expression of hsa-miR-199a-3p influences energy homeostasis and metabolic adaptation via the suppression of IGF1R (insulin-like growth factor 1 receptor) and HIF1A (hypoxia-inducible factor 1-alpha), which are associated with metabolic syndrome [66]. SIRT1 is a key regulator of energy metabolism and adipogenesis. miR-34a-3p suppresses SIRT1, promoting fat accumulation and lipogenesis by inhibiting the PPARγ pathway. This mechanism may explain the observed triglyceride levels in this study. Increased levels of miR-34a-3p in obesity are linked to reduced SIRT1, insulin resistance, and metabolic disorders [2,67]. hsa-miR-143-3p affects insulin signaling by targeting IRS1 (insulin receptor substrate 1) and AKT1, while hsa-miR-124-3p modulates adipocyte differentiation and inflammatory responses through FOXO1 and MAPK14 suppression [68,69]. Similarly, hsa-miR-218-1-3p impairs energy metabolism by suppressing SH2B1 and SOCS3, which are crucial in leptin signaling [70]. Other miRNAs, including hsa-miR-519b-3p, hsa-miR-384, hsa-miR-488-3p, and hsa-miR-877-3p, target the key molecules involved in glucose metabolism, insulin resistance, and adipose tissue differentiation, reinforcing their role in obesity pathophysiology [71,72,73].

These findings align with previous studies, reinforcing the importance of miRNAs in obesity pathophysiology. The identified miRNAs target key regulatory pathways, including Wnt/β-catenin, PPARγ, PI3K/AKT, and IGF-1 signaling, influencing lipid metabolism and inflammation. Table 8 presents the target genes, functions, and related pathways of these miRNAs, emphasizing their significance in obesity-related complications.

## 5. Conclusions

This study identified significant clinical and molecular differences in obese and pre-obese individuals, highlighting the crucial role of gender in obesity. The observed alterations in miRNA expression suggest that these molecules may serve as potential biomarkers, offering new insights into the molecular mechanisms underlying obesity-related metabolic dysfunctions.

Clinically, obesity was associated with significant changes in BMI, lipid profiles, and heightened inflammatory responses, reinforcing its impact on overall health. These physiological alterations align with molecular findings, where the most striking discovery was the statistically significant change in hsa-miR-148a-3p, which is a key regulator of energy metabolism and adipogenesis. This suggests that miRNA dysregulation plays a crucial role in obesity pathogenesis.

Beyond hsa-miR-148a-3p, several other miRNAs—including hsa-miR-503-3p, hsa-miR-34a-5p, hsa-miR-199a-3p, hsa-miR-34a-3p, hsa-miR-143-3p, hsa-miR-124-3p, hsa-miR-218-1-3p, hsa-miR-519b-3p, hsa-miR-384, hsa-miR-488-3p, and hsa-miR-877-3p—were found to play key roles in obesity pathophysiology. Notably, the evaluation of hsa-miR-384, hsa-miR-488-3p, and hsa-miR-877-3p regarding obesity for the first time is particularly significant, as it expands our understanding of the miRNA-mediated regulation of metabolic pathways.

These findings emphasize the potential of miRNA-targeted therapeutic strategies, paving the way for novel approaches to obesity prevention and treatment. By further validating the roles of these miRNAs, future studies can help establish their clinical significance and therapeutic applicability in managing obesity-related metabolic disorders.

Overall, by examining miRNA expression changes across obese, pre-obese, and control groups, this study provides crucial insights into the molecular mechanisms of obesity. The regulatory influence of miRNAs on metabolic processes underscores their importance in both disease progression and potential treatment strategies, marking them as valuable targets for future research and therapeutic interventions.

## Figures and Tables

**Table 1:** Comparison of obese, pre-obese, and control groups.

	Obese (Mean ± SD)	Pre-Obese (Mean ± SD)	Control (Mean ± SD)	*p*-Value
Age, years	36.13 ± 13.29	44.63 ± 15.33	43.53 ± 18.61	0.085
Weight, kg	123.2 ± 22.51 ^a^	79.87 ± 9.63 ^b^	64.6 ± 9.64 ^c^	<0.0001 *
Height, cm	168.17 ± 10.88	170 ± 9.50	171.20 ± 11.19	0.536
BMI, kg/m^2^	43.72 ± 7.07 ^a^	27.52 ± 1.50 ^b^	21.85 ± 1.45 ^c^	<0.0001 *
HDL, mg/dL	37.64 ± 10.78	40.43 ± 14.48	44.61 ± 13.11	0.114
LDL, mg/dL	117.7 ± 34.64	116.22 ± 31.73	104.48 ± 31.07	0.231
CHOL, mg/dL	184.23 ± 35.32 ^a^	168.93 ± 37.65 ^a^	141.27 ± 41.31 ^b^	<0.0001 *
Triglyceride, mg/dL	186.03 ± 90.96 ^a^	158.77 ± 61.47 ^ab^	129.93 ± 64.88 ^b^	0.016 *
Hemoglobin, g/dL	13.84 ± 1.82	13.68 ± 1.58	13.71 ± 2.11	0.936
Neutrophil, mL	5623.67 ± 2139.73	5207.33 ± 2596.09	5113.33 ± 2427.94	0.681
Lymphocyte, mcL	3188.67 ± 1057.27 ^a^	2404.67 ± 895.57 ^b^	2146 ± 1179.12 ^b^	0.001 *
Monocyte, mcL	644.33 ± 219.23	628.3 ± 246.15	628.67 ± 256.36	0.958
Platelet, mcL	295,766.67 ± 77,440.38	274,866.67 ± 94,739.47	281,473.33 ± 124,724.71	0.715

The sample numbers of the groups are as follows: obese group (*n* = 30), pre-obese group (*n* = 30), and control group (*n* = 26). Statistical analyses were performed according to these sample sizes. Different superscript letters (a, b, c) within a row indicate statistically significant differences between groups (* *p* < 0.05).

**Table 2:** Statistical analysis of obese patients (obese plus pre-obese) vs. controls.

Position	miRNA ID	Fold Regulation	*p*-Value
22	hsa-miR-148a-3p	−2.09	<0.0001
43	hsa-miR-503-3p	−2.16	0.011

**Table 3:** Statistical analysis of obese group vs. control group.

Position	miRNA ID	Fold Regulation	*p*-Value
5	hsa-miR-34a-5p	−2.03	0.007
9	hsa-miR-199a-3p	−2.09	0.005
22	hsa-miR-148a-3p	−2.39	0.005
23	hsa-miR-34a-3p	−2.36	0.006
57	hsa-miR-143-3p	−2.69	0.017

**Table 4:** Statistical analysis of pre-obese group vs. control group.

Position	miRNA ID	Fold Regulation	*p*-Value
77	hsa-miR-124-3p	−2.20	0.035

**Table 5:** Statistical analysis of obese women vs. obese men.

Position	miRNA ID	Fold Regulation	*p*-Value
63	hsa-miR-218-1-3p	−3.04	0.034

**Table 6:** Statistical analysis of male obese group (obese plus pre-obese men) vs. male controls.

Position	miRNA ID	Fold Regulation	*p*-Value
29	hsa-miR-519b-3p	3.23	0.040
15	hsa-miR-384	−4.75	0.013
16	hsa-miR-488-3p	−3.45	0.006
22	hsa-miR-148a-3p	−2.23	0.007
23	hsa-miR-34a-3p	−2.18	0.006
34	hsa-miR-877-3p	−2.08	0.020

**Table 7:** Statistical analysis of female obese group (obese plus pre-obese women) vs. female controls.

Position	miRNA ID	Fold Regulation	*p*-Value
43	hsa-miR-503-3p	−2.33	0.032

**Table 8:** Target genes and related pathways for miRNAs.

miRNA	Target Gene	Function	Related Pathway
hsa-mir-148a-3p	PPARGC1A, FTO, LEP	Regulates PPARGC1A in adipogenesis, modulates leptin signaling, and influences energy metabolism.	Wnt/β-catenin, TGF-β signaling pathway
hsa-mir-503-3p	VEGFA, FGF2	Interact with vascular responses and tissue oxygenation by suppressing VEGFA.	IGF-1 and angiogenesis signaling pathway
hsa-mir-34a-5p	SIRT1, PPRAG	Enhances lipogenesis and inflammation by suppressing SIRT1; regulates PRAG	P53 PPAR signaling pathway
hsa-mir-199a-3p	HIF1A, IGF1R	Regulates energy homeostasis and metabolic adaptation by suppressing HIF1A and IGF1R.	mTOR hypoxia and IGF-1 signaling pathway
hsa-mir-34a-3p	SIRT1, PPRAG	Increases fat accumulation by suppressing SIRT and supports inflammation.	Lipid metabolism, PPARγ
hsa-mir-143-3p	AKT1, IRS1	Regulates insulin signaling by targeting IRS1 and AKT1 and interacts with glucose uptake.	PI3K/AKT, signaling pathway
hsa-mir-124-3p	FOXO1, MAPK14	Enhances adiposity differentiation. It is related to inflammation and metabolic stress.	JAK/STAT, MAPK
hsa-mir-218-1-3p	SH2B1, SOCS3	Decreases energy consumption by suppressing SH2B1 in leptin signaling.	Leptin-S TAT3 signaling pathway
hsa-mir-519b-3p	TP53, CCND1	Related to cellular stress due to obesity through targeting cell cycle regulatory genes.	Cell cycle and apoptosis signaling pathway
hsa-mir-384	IRS2, PIK3R1	Interact with glucose metabolism by suppressing regulatory genes of insulin signaling.	PI3K/AKT, insulin signaling pathway
hsa-488-3p	FOXO1, LEPR	Regulates leptin receptors and influences energy metabolism; it also suppresses FOXO1.	Nervous system signaling pathway
hsa-mir-877-3p	IGFR1, IRS1	Impairs insulin resistance and glucose metabolism by suppressing IGFR1 and IRS1.	IGF-1 and insulin signaling pathway

## Data Availability

All data used in this study can be obtained from the authors if necessary.

## Supplementary Materials

The following supporting information can be downloaded at https://www.mdpi.com/article/10.3390/cimb47040280/s1.
